# Effects of the dietary approach to stop hypertension (DASH) diet on blood pressure, blood glucose, and lipid profile in adolescents with hemophilia: A randomized clinical trial

**DOI:** 10.1002/fsn3.1972

**Published:** 2020-10-29

**Authors:** Atena Mahdavi, Hamed Mohammadi, Sahar Foshati, Nafiseh Shokri‐Mashhadi, Cain C. T. Clark, Alireza Moafi, Mohammad Hossein Rouhani

**Affiliations:** ^1^ Food Security Research Center and Department of Community Nutrition School of Nutrition and Food Science Isfahan University of Medical Sciences Isfahan Iran; ^2^ Food Security Research Center and Department of Clinical Nutrition School of Nutrition and Food Science Isfahan University of Medical Sciences Isfahan Iran; ^3^ Centre for Intelligent Healthcare Coventry University Coventry UK; ^4^ Pediatric Hematology and Oncology Isfahan University of Medical Sciences Isfahan Iran

**Keywords:** adolescents, blood glucose, blood pressure, dietary approach to stop hypertension, hemophilia, lipid profile

## Abstract

Children with hemophilia are an enhanced risk of modifiable cardiovascular and metabolic abnormalities. There is currently no nutritional guideline to prevent or manage cardiometabolic risk factors in these patients. Therefore, the present study sought to investigate the effect of the Dietary Approaches to Stop Hypertension (DASH) diet on cardiovascular and metabolic risk factors among children with hemophilia. In this parallel randomized clinical trial, 40 children (all male) with hemophilia were randomly allocated to the DASH group (*n* = 20) or control group (*n* = 20) for 10 weeks. The intervention group received the DASH diet (50%–55% of energy from carbohydrate, 27%–30% of energy from fat and 16%–18% energy from protein), and the control group received nutritional recommendations based on healthy eating practices. Systolic blood pressure (SBP), diastolic blood pressure (DBP), fasting blood sugar (FBS), triglyceride (TG), total cholesterol (TC), low‐density lipoprotein (LDL), and high‐density lipoprotein (HDL) were measured at the beginning and end of the study. Serum vitamin C was measured as a biomarker of compliance with the DASH diet. Study was registered at IRCT.ir (IRCT20130903014551N6). A significant increase in serum vitamin C in the DASH diet group was observed compared to the control group (*p* = .001), indicating good compliance with the DASH diet. There was a significant reduction in SBP (−0.48 mmHg), DBP (−0.48 mmHg), FBS (−5.86 mg/dl), TC (−16.07 mg/dl), TG (−17.21 mg/dl), and LDL‐C (−9.79 mg/dl), and a significant increase in HDL‐C (3.39 mg/dl), in the DASH diet group compared with the control group. Adherence to the DASH diet in children with hemophilia yielded beneficial effects in blood pressure, lipid profiles, and FBS.

## Graphical abstract

Forty adolescents with hemophilia were assigned to the DASH group or control group. After 10 weeks, beneficial changes were observed in blood pressure, lipid profile, and FBS among adolescents in the DASH group.

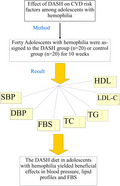

## 1 INTRODUCTION

Hemophilia is a rare X‐linked congenital bleeding disorder characterized by late blood coagulation with excessive hemorrhage, frequently into joints, due to deficiencies in clotting factors VIII (FVIII) and IX (FIX) (Zimmerman & Valentino, 2013). Management of bleeding in patients with hemophilia involves replacing the clotting factor, or the use of blood products as a cheaper alternative in the circulation, and the initiation of prophylactic infusions of clotting factor (Singleton et al., 2010).

In contemporary practice, hemophilia management has improved, such that patients are now living longer with lower infectious complications, which has led to the development of age‐related complaints in this population (Philipp, 2010). As a result, the risk of general health problems, such as obesity, and its complications and comorbidities, can be increased (Wong et al., 2011). Moreover, there is a growing number of studies reporting that the prevalence of cardiovascular risk factors, including hypertension, hypercholesterolemia, and diabetes, is high in these patients and may, therefore, influence general health and the cost of care (Alperstein et al., 2018). Recent data, from the 2012 National Health Cost and Utilization Project database, prevalence of hypertension among hemophilic children is 67% higher than general pediatric population (Alperstein et al., 2018). In addition, evaluation of fasting glucose and insulin levels in children and young adults with hemophilia has also demonstrated that fasting blood glucose levels are significantly higher than matched, otherwise healthy, controls (Yıldız et al., 2019). Furthermore, a large US cohort study revealed that hyperlipidemia was more prevalent in the hemophilia population (15.9%) compared with healthy subjects (11.9%) (Pocoski et al., 2014). Therefore, urgent attention must be given to the management and prevention of undesirable cardio metabolic risk factors in patients with hemophilia.

Despite the high prevalence of modifiable cardiovascular and metabolic risk factors in patients with hemophilia, current guidelines for hemophilia management do not adequately address the cardiovascular risk prevention approaches (Stoffman et al., 2019). Long‐term lifestyle modification is purported to represent the best preventative measure of cardiovascular risk factors in patients with hemophilia. This can be achieved by following the guidelines for the management of cardiovascular risk factors in the general population (Sousos et al., 2016). Dietary behavior in patients with hemophilia is beneficial and can protect their health and prevent nutritional diseases (Chinnappa et al., 2020; Czepa et al., 2014). In this regard, a recent meta‐analysis study advocated the Dietary Approach to Stop Hypertension (DASH) as the safest and most effective nutritional strategy to improve systolic and diastolic blood pressure, in addition to significant reductions in total cholesterol and low‐density lipoprotein (LDL) levels in the general population (Siervo et al., 2015). Furthermore, the promising role of the DASH diet in improving fasting insulin levels and fasting blood glucose has been previously shown (Akhlaghi, 2020). Although some previous evidence has asserted that the origins of cardiovascular disease (CVD) begins in childhood, there are limited recommendations available to improve diet intake and modify blood pressure, blood glucose, and lipid profile in adolescents with hemophilia. Therefore, the present study sought to investigate the effect of the DASH diet on cardiovascular and metabolic risk factors among adolescents with hemophilia.

## 2 MATERIAL AND METHODS

### 2.1 Participants

The present parallel randomized clinical trial was performed from March to June 2020. Subjects with hemophilia were recruited from Omid Hospital, Isfahan, Iran. Volunteers were eligible if they (a) were male, (b) were between 10 to 18 years old, (c) had clotting factor (VII, VIII and IX) deficiency, (d) have not used antioxidant supplements within the previous 3 months, and (e) were not on a specific or prescribed diet. Low compliance with dietary intervention and incidence of a new chronic disease during the study were determined as exclusion criteria.

Sample size was determined by *n* = 2[(Z_1‐α/2_ + Z_1‐β_)^2^ × S^2^]/ Δ^2^ where α = 0.05 (type one error) and β = 20% (type two error). Total cholesterol (TC) was considered as the main variable. A previous study demonstrated that the standard deviation of TC in hemophilia patients was 5.3 mg/dl (Özdemir et al., 2020), and the minimal detectable difference of TC was 5 mg/dl. Accordingly, the required sample size was 36 for this study. To account for incidence of attrition, 40 hemophilia adolescents were recruited for the current clinical trial. An introductory meeting was set up to explain the aims and details regarding the study, and subsequently, prior to study commencement, adolescents and one of their parents signed informed written assent/consent forms. The Research Council and Ethical Committee of “blinded for peer review” University of Medical Sciences, “blinded for peer review,” “blinded for peer review” and “blinded for peer review” approved this study (Code: “blinded for peer review”). This randomized clinical trial was registered at “blinded for peer review.”

### 2.2 Study procedure and dietary intervention

Adolescents were randomly allocated to the DASH (*n* = 20) or control group (*n* = 20) for 10 weeks. We assigned a code to each child and used SPSS 20 (IBM) for randomization. In the DASH group, total energy expenditure was individually calculated using Harris–Benedict equation. The DASH diet was administered based on a previous modified DASH diet for adolescents (Couch et al., 2008). Macronutrients distribution was as follows: 50%–55% of energy from carbohydrate, 27%–30% of energy from fat and 16%–18% energy from protein. Daily food menus were designed by emphasizing on consumption of whole grains, fruits and vegetables, legumes, nuts, seeds, low‐fat dairy, and white meat. Also, consumption of red meat and sodium was restricted (Table 1). Daily food menus were explained and taught to adolescents and their parents. Patients in the control group received nutritional recommendations, based on healthy eating, including chewing food completely, using low‐volume frequent meals, limiting added fat and sugar, using healthy snacks, drinking adequate water and avoiding deep frying. Previous studies showed that serum vitamin C is a viable biochemical indicator of complying with the DASH diet (Saneei et al., 2014); so, compliance with the DASH diet was evaluated by measuring serum vitamin C at baseline and after 10 weeks of intervention. Adolescents and their parents participated in meetings scheduled at baseline and 2‐, 4‐, 6‐ and 8‐weeks, whilst parents additionally completed a one‐day food record on the 1st, 5th and 10th week of the study (three in total). All collected food records were analyzed by Nutritionist IV using the USDA database.

**TABLE 1:** A sample menu of the prescribed diet to the DASH group (6,276 kJ (1,500 kcal), 55% from carbohydrate, 17% from protein, 28% from fats)

Food groups	Serving size	Food item (g/d)
Grains	6	Whole‐Bread (60)
Vegetables	5	Whole‐Cereals (150)
Fruits	5	Cooked beans (32)
Dairy	2	Cooked green beans (42)
Meat	2.5	Lettuce (50)
Nuts/Seeds	1	Tomato (60)
Fats/Oils	6	Cucumber (100)
		Cooked carrots (67)
		Apple (200)
		Peach (185)
		Banana (64)
		Low‐fat milk (230)
		Low‐fat yogurt (230)
		Low‐fat cheese (15)
		Cooked chicken (40)

### 2.3 Blood pressure measurement

Blood pressure was measured, with participants in a seated position, after 10 min of rest, using a standard mercury sphygmomanometer. The systolic blood pressure (SBP) was determined by the first sound (Korotkoff phase 1), and diastolic blood pressure (DBP) was determined as a fade of the sound (Korotkoff phase 5). The average of two manual measurements was recorded.

### 2.4 Biochemical assessment

A blood specimen was taken after 12 hr of fasting in the early morning. After clotting, samples were centrifuged at 3,000 × g for 10 min to separate the serum. The concentration of blood glucose was measured by an enzymatic colorimetric method based on activity of glucose oxidase enzyme (Pars Azmoon, Tehran, Iran), and serum concentration of triglyceride (TG) and TC was evaluated by enzymatic colorimetric method. After blocking low‐density lipoprotein (LDL), very low‐density lipoprotein, and chylomicrons by antibodies, the concentration of high‐density lipoprotein (HDL) was measured by photometric method. Similarly, enzymatic colorimetric tests were utilized to measure LDL, after blocking HDL, VLDL and chylomicrons.

### 2.5 Statistical analysis

The analyses were performed on the basis of an intention‐to‐treat (ITT) approach. Missing values were treated according to linear regression method (Xi et al., 2018). Kolmogorov‐Smirnov test and Q‐Q plot were used to evaluate normal distribution, and results showed that TG was not normally distributed; therefore, it was log transformed. Chi‐square tests were used to compare qualitative variables between the DASH and control group, and quantitative variables were compared between two groups using independent Student's *t*‐test. To adjust for confounding variables (baseline values), we conducted analysis of covariance (ANCOVA). Data were presented as mean and standard deviation, and all data analyses were conducted using SPSS version 20 statistical software.

### 2.6 Results

**FIGURE 1:**
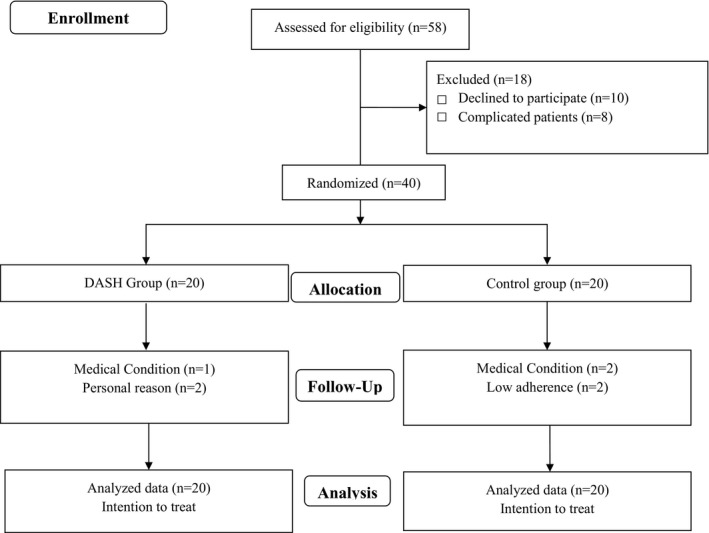
Flow Diagram illustrating participant selection process and study procedure

Among the 40 subjects with hemophilia who enrolled in the study, three patients in the DASH diet group (due to medical condition and personal reasons) and four patients in the control group (due to medical condition and low adherence to intervention) were withdrawn from study (Figure 1). The analyses were performed according to ITT approach, and thus, all 40 participants were enrolled in the final analyses. There were no side effects following DASH diet among the participants.

Table 2 indicates baseline characteristics of study participants in each group. There were no significant differences between the two groups in terms of age, BMI percentile, and WC. However, at baseline, individuals in the DASH diet group had a higher weight and height percentile, compared with those in control group.

**TABLE 2:** Baseline characteristic of study subjects

Variable^a^	DASH diet (*n* = 20)	Control diet (*n* = 20)	*p* ^b^
Age (year)	13.85 ± 2.64	14.8 ± 2.74	.30
Deficient Factor			
Factor VII (%)	55	60	.562
Factor IX (%)	35	20	
Other Factors (%)	10	20	
Weight (percentile)	76.02 ± 25.66	73.98 ± 16.04	.03
Height (percentile)	50.75 ± 34.48	39.46 ± 23.69	.006
Percentile BMI	9.2 ± 0.81	8.94 ± 0.93	.88
WC (cm)	83.25 ± 15.1	79.2 ± 9.11	.07

BMI; body mass index, WC; waist circumference.

^a^ Variables are expressed as mean ± *SD*.

^b^ *p*‐values resulted from independent *t* tests for quantitative and chi‐square for qualitative variables between the two groups.

Dietary intakes of study participants are presented in Table 3. Based on food diaries, the mean intake of energy, carbohydrate, protein, and fat during the trial were not significantly different between groups. As expected, dietary intake of sodium was significantly lower (1,172.35 vs. 1,580.94 mg/day, *p* = .003), while calcium intake was higher (754.26 vs. 545.28 mg/day, *p* = .01) in the DASH versus control group. Individuals in the DASH group tended to have higher intakes of potassium (1987.52 vs. 1624.36 mg/day, *p* = .07) and vitamin C (72.55 vs. 54.43 mg/day, *p* = .15), compared with those in the control group.

**TABLE 3:** Dietary intake of the study participants throughout the study^a^

Variable	DASH diet (*n* = 20)	Control diet (*n* = 20)	*p* ^b^
Energy (Kcal/day)	1,144.58 ± 154.65	1,293.71 ± 310.73	.08
Carbohydrate (g/day)	167.75 ± 32.89	173.84 ± 32.89	.57
Protein (g/day)	49.74 ± 18.59	50.71 ± 18.59	.87
Fat (g/day)	41.08 ± 10.41	41.11 ± 10.41	.99
Sodium (mg/day)	1,172.35 ± 398.36	1,580.94 ± 398.36	.003
Vitamin A (RE/day)	613.74 ± 383.34	501.35 ± 383.34	.37
Vitamin E (mg/day)	14.69 ± 6.74	12.46 ± 6.74	.31
Vitamin C (mg/day)	72.55 ± 38.66	54.43 ± 38.66	.15
Vitamin K (ug/day)	35.08 ± 17.96	26.89 ± 17.96	.16
Vitamin B1 (mg/day)	0.9 ± 0.22	0.83 ± 0.22	.42
Vitamin B2 (mg/day)	1.38 ± 0.35	1.23 ± 0.35	.22
Vitamin D (ug/day)	1.91 ± 1.38	0.75 ± 1.38	.01
Potassium (mg/day)	1987.52 ± 574.08	1642.36 ± 574.08	.07
Calcium (mg/day)	754.26 ± 246.96	545.28 ± 246.96	.01
Selenium (mg/day)	0.08 ± 0.08	0.07 ± 0.08	.63
Zinc (mg/day)	6.11 ± 1.78	5.52 ± 1.78	.31
Iron (mg/day)	8.48 ± 3.08	8.66 ± 3.08	.86
Dietary Fiber (g/day)	14.43 ± 4.42	12.10 ± 4.42	.11
Soluble Fiber (g/day)	0.52 ± 0.17	0.38 ± 0.17	.02

^a^ Variables are expressed as mean ± *SD*.

^b^ Obtained from independent *t* test

**FIGURE 2:**
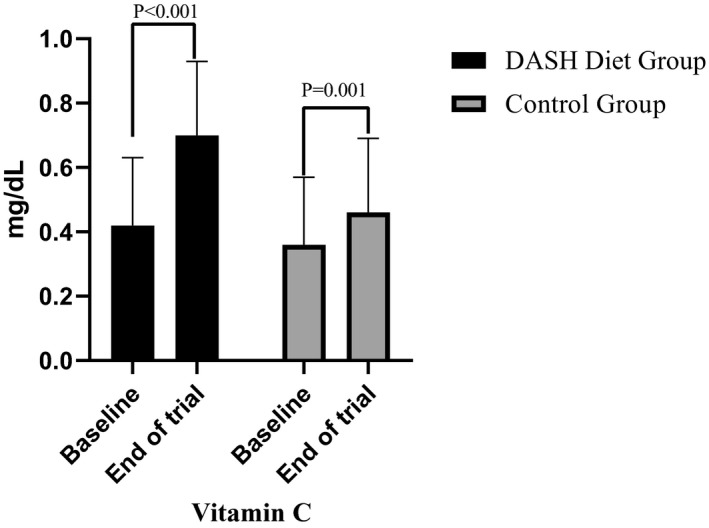
Serum vitamin C levels at baseline and end of trial in each group

Figure 2 presents serum vitamin C levels at baseline and end of trial in each group. At the end of trial, serum vitamin C levels were significantly increased in the DASH diet group (0.28 mg/dl) and control group (0.10 mg/dl) compared to baseline values. Between‐group comparison further indicated a significant rise in serum vitamin C levels in the DASH diet group compared to control group (*p* = .001), indicating good compliance of the participants to the DASH diet.

As demonstrated in Table 4, within group comparison showed a significant reduction in SBP (−0.48 mmHg), DBP (−0.48 mmHg), FBS (−5.86 mg/dl), TC (−16.07 mg/dl), TG (−17.21 mg/dl), and LDL‐C (−9.79 mg/dl) in the DASH diet group. Also, a significant rise was seen regarding HDL‐C (3.39 mg/dl) in the DASH diet group. Compared with the control group, adherence to the DASH diet led to significant reduction in SBP (*p* = .04), FBS (*p* = .004), TC (*p* = .001), TG (*p* = .04), and LDL‐C (*p* = .001), and a significant rise in HDL‐C (*p* = .01). There were no significant differences regarding DBP between groups.

**TABLE 4:** The effects of DASH diet on lipid profile, fasting blood sugar, and blood pressure^a^

	DASH Diet (*n* = 20)				Control Diet (*n* = 20)				*p* ^c^
	Baseline	End of trial	Change	P^b^	Baseline	End of trial	Change	*p* ^b^	
SBP (mmHg)	12.00 ± 1.21	11.51 ± 0.90	−0.48 ± 0.59	0.002	12.00 ± 0.97	12.10 ± 0.61	0.10 ± 0.79	.54	<.001
DBP (mmHg)	8.15 ± 0.98	7.66 ± 0.68	−0.49 ± 0.49	<0.001	8.1 ± 0.78	8.15 ± 0.55	0.05 ± 0.69	.70	0.96
FBS (mg/dl)	96.00 ± 9.95	90.13 ± 7.44	−5.86 ± 5.70	<0.001	95.2 ± 8.81	94.39 ± 8.18	−0.80 ± 6.00	.55	.004
TC (mg/dl)	155.85 ± 18.81	139.77 ± 18.97	−16.07 ± 10.43	<0.001	95.2 ± 8.81	134.96 ± 25.40	−0.13 ± 11.78	.95	.001
TG (mg/dl)	114.45 ± 54.50	97.23 ± 44.86	−17.21 ± 20.1	0.001	113.8 ± 41.80	110.74 ± 49.08	−3.00 ± 15.65	0.18	.04
HDL‐c (mg/dl)	43.60 ± 7.17	46.99 ± 7.55	3.39 ± 4.14	0.002	39.75 ± 7.87	40.43 ± 7.75	0.68 ± 2.56	.26	.01
LDL‐c (mg/dl)	88.40 ± 19.41	78.6 ± 15.13	−9.79 ± 12.53	0.002	92.65 ± 25.84	94.42 ± 23.94	1.77 ± 12.12	.52	.001
Vitamin C (mg/dl)	0.42 ± 0.21	0.70 ± 0.23	0.28 ± 0.19	<0.001	0.36 ± 0.21	0.46 ± 0.23	0.10 ± 0.15	.007	.001

Abbreviations: DBP, diastolic blood pressure;FBS, fasting blood glucose; HDL‐c, high‐density lipoprotein cholesterol; LDL‐c, low‐density lipoprotein cholesterol; SBP, systolic blood pressure; SGOT, serum glutamic oxaloacetic transaminase; TC, total cholesterol; TG, triglycerides.

^a^ Variables are expressed as mean ± *SD*.

^b^ Obtained from paired *t* test.

^c^ Obtained from ANCOVA, adjusted for baseline value.

## 3 DISCUSSION

Dietary modification represents a promising intervention for the management of metabolic risk factors, such as hypertension, hyperglycemia, and dyslipidemia, in many chronic diseases. Nevertheless, there is no specific dietary recommendation, for patients suffering from hemophilia, to improve their metabolic profile. Indeed, a distinct dearth of nutritional interventions has been carried out in patients with hemophilia; therefore, we sought to assess the effect of the DASH diet, as a healthy dietary pattern, on metabolic indicators of adolescents with hemophilia. According to our findings, compliance with the DASH diet, as compared to reception of healthy dietary recommendations, could lead to significant reductions in SBP, FBS, TG, TC, and LDL‐C, and increases in HDL‐C, after 10 weeks, in adolescents with hemophilia. However, no significant between‐group differences were found for DBP during the study.

### 3.1 Blood pressure

In contrast to the control group, both SBP and DBP were significantly reduced in the intervention group. Nonetheless, between‐group differences were significant for SBP, but not for DBP. It seems that SBP had a stronger response to dietary sodium and potassium, two nutrients modified in DASH diet, compared with DBP (Mente et al., 2014). Therefore, it seems that the DASH diet may be more effective for the reduction of SBP than DBP in normotensive adolescents with hemophilia. In the current evidence base, there is a distinct lack of clinical trials to have investigated the effect of the DASH diet on blood pressure in adolescents. Some of these trials have confirmed our findings, and reported a significant reduction in SBP (Couch et al., 2008); although some others have contradicted our findings and reported a significant reduction in DBP (Saneei et al., 2013). The discrepancy in such findings may be attributed to differences in methodologies. Indeed, this issue was also mentioned by Bricarello et al. (2018) who recently conducted a systematic review to evaluate the impact of the DASH diet on childhood blood pressure, but could not perform a meta‐analysis. Interestingly, our findings seem to be in line with the findings of similar randomized clinical trials in adults. When the results of clinical trials conducted on adult were pooled in a recent meta‐analysis, it was found that the DASH diet can elicit a greater reduction in SBP, as compared to DBP (Siervo et al., 2015). It was also suggested that DBP is mainly reduced by antihypertensive medications, rather than dietary modifications (Neter et al., 2003). In addition, it was indicated that reductions in SBP, as well as DBP, can be more pronounced in individuals with higher blood pressure or higher BMI at baseline (Siervo et al., 2015). Since our subjects did not take antihypertensive medications and had normal blood pressure and BMI values, it is not surprising that following the DASH diet elicited a small, but significant, reduction only in SBP. Nevertheless, it has been shown that even a minor reduction in SBP can effectively decrease vascular mortality (Prospective Studies Collaboration, 2002).

The DASH diet is rich in vegetables, fruits, nuts, legumes, whole grains, white meats, and dairy products. As a result, it is comprised of numerous blood pressure‐lowering nutrients, such as potassium, nitrate, magnesium, calcium, folate, vitamin C, fiber, and amino acids like arginine (Saneei et al., 2014). However, the DASH diet limits the intake of salt and processed foods, accordingly, it is low in sodium which is a nutrient linked with increasing blood pressure (Bazzano et al., 2013). According to recent meta‐analyses, the DASH diet appears to be the most effective dietary modification for reducing blood pressure (Schwingshackl et al., 2019). Moreover, it seems that this diet may decrease blood pressure through increased renal sodium excretion (Akita et al., 2003), reduction of inflammatory biomarkers (Soltani et al., 2018), elevation of antioxidant capacity (Al‐Solaiman et al., 2009), and improved endothelial function by means of enhanced nitric oxide bioavailability (Lin et al., 2012).

### 3.2 Blood glucose

During our study, FBS was significantly decreased in the intervention group, but remained statistically congruent in the control group. As a consequence, the between‐group difference in FBS was significant at the end of 10 weeks. Contrary to this finding, Saneei et al. (2013) did not find any significant changes in FBS, after 6 weeks of intervention with the DASH diet, in children suffering from metabolic syndrome. It is conceivable that the contradiction between the present study and (Saneei et al., 2013) may be attributable to, first, the compliance of participants to the DASH diet being higher in the present study, and second, the duration of our trial was longer. Indeed, the length of intervention, that is, adherence to the DASH diet, has been shown to modify its effects on the glucose‐insulin control system (Shirani et al., 2013). It is worth to mention that the results of similar trials conducted in adults with good dietary compliance during long‐term periods were in agreement with our result regarding FBS (Azadbakht et al., 2005).

The DASH dietary pattern can be considered as both a low glycemic index and low energy dense diet. Foods with low glycemic indices tend to lower postprandial glucose responses through the slow and sustained release of monosaccharides from the gastrointestinal tract into the blood circulation (Radulian et al., 2009). Foods with low energy densities tend to protect against weight‐associated insulin resistance through increased satiety and decreased subsequent food intake (Rolls et al., 2005). It is also possible that the DASH diet results in improved insulin sensitivity, independent of weight changes, through its high content of beneficial micronutrients, such as magnesium and folate (Ard et al., 2004). Indeed, magnesium is crucial to insulin secretion by pancreatic β‐cells, interaction between insulin and its receptor, and post‐receptor insulin signaling which involves tyrosine kinase mediated phosphorylation (Simental‐Mendía et al., 2016). Folate is essential to prevent hyperhomocysteinemia, which causes insulin resistance by inducing endoplasmic reticulum stress and provoking inflammation in adipose tissue (Lind et al., 2019). Overall, the DASH diet seems to be a good choice for the improvement of glycemic control.

### 3.3 Lipid profile

In our study, lipid profile, including TG, TC, LDL‐C, and HDL‐C, was significantly ameliorated in the intervention group, and remained statistically unchanged in the control group. As a result, analyses of between‐group differences revealed that the DASH diet can reduce TG, TC, and LDL‐C, and increase HDL‐C, in adolescents with hemophilia. Contrary to our findings, Saneei et al. (2013) reported that the DASH diet did exert any significant effect on the lipid profile of children with metabolic syndrome. As we alluded to above, in Saneei et al, the trial was short‐term and suffered from relatively poor dietary compliance.

In general, our findings seem to be concordant with the findings of similar trials in adults. In a recent meta‐analysis of these trials, pooled results showed that the DASH diet can decrease TG, TC, and LDL‐C, and increase HDL‐C. Nevertheless, these pooled results reached statistical significance for TC and LDL‐C but not for TG and HDL‐C. The pooled results of TG and HDL‐C appear to be respectively confounded by significant publication bias and significantly high between‐study heterogeneity (Siervo et al., 2015). Therefore, the pooled results of TG and HDL‐C may not be adequately reliable. It is important to note that significant decrements in TG, and significant increments in HDL‐C, were observed following intervention with the DASH diet in well‐designed and well‐conducted trials (Azadbakht et al., 2005).

Although the DASH diet is low in total fat, saturated fat, and cholesterol, the beneficial effects of this diet on lipid profile are not only due to its low‐fat content (Miller et al., 2006). Indeed, vegetables and dairies, which are two indispensable constituents of the DASH diet, can also help to improve lipid profile. Intake of vegetables that are rich in dietary fiber has been reported to be inversely associated with dyslipidemia, characterized by high TC and high LDL‐C (Song et al., 2016). Dietary fiber can also increase the excretion of bile acids in feces, reduce the cholesterol pool in the liver, modify the activity of enzymes involved in cholesterol homeostasis, up‐regulate the expression of hepatic LDL‐C receptors, and increase plasma LDL‐C removal (Papathanasopoulos & Camilleri, 2010). Concomitantly, intake of dairy products that are rich in calcium has been reported to be inversely associated with dyslipidemia characterized by high TG and low HDL‐C (Song et al., 2016). Calcium can reduce fat absorption via the formation of insoluble calcium‐fatty soaps, affect the rate of lipolysis and lipogenesis via suppression of calciotropic hormones, modulate the activity of microsomal triglyceride transfer protein, and increase thermogenesis (Heshmati et al., 2019).

### 3.4 Vitamin C

In our study, vitamin C increased in the intervention group compared to the control group. Vitamin C is a water‐soluble antioxidant; therefore, the intake of fruits and vegetables in the DASH diet can increase vitamin C and benefit hemophilia patients. Previous studies showed that prevention of oxidative stress is important for protein folding in patients with hemophilia (Malhotra et al., 2008). Therefore, it is adherence to DASH diet may have long‐term favorable effects on chronic conditions in patients with hemodialysis.

### 3.5 Strengths and limitations

The main strength of our study is that dietary compliance was assessed by a valid and reliable biomarker, that is, serum levels of vitamin C, allowing us to assert veracity in our findings. Previous studies showed that urinary sodium and potassium excretion, as measured in a 24‐hr urine sample, can be as a biomarker of compliance with the DASH diet. Nevertheless, collecting of 24‐hr urine sample is difficult. Also, day to day variation of about 24‐hr urinary sodium is high, and several samplings are needed to eliminate day to day variation. (Taylor et al., 2010). Since it was not applicable for children, we used vitamin C because it does not have these limitations. The main limitation of our study is that blinding was not applicable due to the nature of our intervention.

## 4 CONCLUSION

The DASH diet was primarily designed to control cardiovascular risk factors in healthy or unhealthy adults. Nevertheless, our findings indicate that this diet can also be beneficial in adolescents with hemophilia. Indeed, our findings suggest that the DASH diet can improve blood pressure, blood glucose, and lipid profile. Therefore, it seems that this dietary approach can viably serve as an effective modifier of metabolic risk factors in adolescents suffering from hemophilia. However, further, well‐designed and well‐conducted, studies are needed to enable firm conclusions to be made.

## 5 TRANSPARENCY DECLARATION

The lead author affirms that this manuscript is an honest, accurate, and transparent account of the study being reported. The reporting of this work is compliant with CONSORT guidelines. The lead author affirms that no important aspects of the study have been omitted and that any discrepancies from the study as planned (The Research Council and Ethical Committee of Isfahan University of Medical Sciences, Isfahan, Iran and Food Security Research Center, Isfahan University of Medical Sciences, Isfahan, Iran approved this study (Code: IR.MUI.RESEARCH.REC.1399.098). This randomized clinical trial was registered at IRCT.ir (IRCT20130903014551N6)) have been explained.

## Data Availability

Data are available on request due to privacy/ethical restrictions.
